# Addition and Subtraction but Not Multiplication and Division Cause Shifts of Spatial Attention

**DOI:** 10.3389/fnhum.2018.00183

**Published:** 2018-05-03

**Authors:** Mengjin Li, Dixiu Liu, Min Li, Wenshan Dong, Yalun Huang, Qi Chen

**Affiliations:** ^1^School of Psychology, South China Normal University, Guangzhou, China; ^2^Center for Studies of Psychological Application, South China Normal University, Guangzhou, China; ^3^Guangdong Key Laboratory of Mental Health and Cognitive Science, South China Normal University, Guangzhou, China; ^4^Department of Psychology, Gannan Medical University, Ganzhou, China

**Keywords:** numerical cognition, mental arithmetic, number and space, target detection task, attention shifts

## Abstract

Many studies have shown that solving addition and subtraction problems can induce overt shifts of spatial attention. In particular, right-side targets are detected faster than left-side targets when preceded by an addition operation, while left-side targets are detected faster than right-side targets when preceded by a subtraction operation. However, the interaction between space and arithmetic in multiplication or division is hardly studied and remains controversial. In order to make a strong case for the interaction between space and mental arithmetic, we attempted to replicate the spatial-arithmetic association in addition and subtraction (Experiment 1), and at the same time investigated whether shift of spatial attention would also be induced by multiplication or division operations (Experiment 2). We found that solving addition problems facilitated the detection of right-side targets, whereas left-side targets were detected faster after solving subtraction problems. However, no interaction between space and arithmetic operation was observed in multiplication or division. The implication of these findings is discussed.

## Introduction

In the past two decades, the relation between number and space has been examined in several studies (e.g., Dehaene et al., 1993; Fischer et al., 2003; Hubbard et al., 2005; Wood et al., 2008; Chen and Verguts, 2010). In a seminal study, Dehaene et al. (1993) found that small numbers are associated with faster left-hand responses and larger numbers with faster right-hand responses (Dehaene et al., 1993). Recently, it has been proposed that arithmetic operations are also associated with space (McCrink et al., 2007; Knops et al., 2009a,b; McCrink and Wynn, 2009; Chen and Verguts, 2012). For example, McCrink et al. (2007) found a systematic bias in non-symbolic addition and subtraction operations, i.e., adults tend to overestimate the results of addition problems and underestimate the subtraction problem results. This bias is called the operational momentum effect (McCrink et al., 2007), which provides evidence for the spatial nature of number processing (McCrink et al., 2007; Knops et al., 2009b). Consistently, Knops et al. (2009b) found that subjects tend to select options on the right upper side after solving addition problems, and options on the left upper side after solving subtraction problems.

More recently, a number of studies have tested the hypothesis that addition and subtraction can cause shifts of spatial attention rightward or leftward, respectively (Masson and Pesenti, 2014; Mathieu et al., 2016; Masson et al., 2017; Liu et al., 2017a,b). Using a target detection task primed by an addition or subtraction operation, it was found that solving subtraction problems facilitates the detection of left-side targets, whereas solving addition problems facilitates the detection of right-side targets (Masson and Pesenti, 2014). Liu et al. (2017a,b) modified the target detection paradigm of Fischer et al. (2003) to reexamine the time course of the interaction between space and arithmetic (in particular, addition and subtraction). Their results confirmed that addition and subtraction can induce horizontal shifts of spatial attention (i.e., right-side targets are detected faster than left-side targets after solving addition problems, while left-side targets are detected faster than right-side targets after solving subtraction problems), and the spatial-arithmetic associations are shown robustly at 300 ms after the arithmetic operations (Fischer et al., 2003; Mathieu et al., 2016; Liu et al., 2017a,b). These findings confirm that there is a close link between elementary arithmetic operations and visuospatial attention orientation (Knops et al., 2009a,b), and there are similarly recruited brain activation patterns of addition/subtraction operation to rightward/leftward eye movement (Knops et al., 2009a). Evidence of the association between arithmetic (addition or subtraction) and space also comes from studies that included motor activity (Hartmann et al., 2015, 2016; Zhu et al., 2017). For example, Zhu et al. (2017) observed that participants move their eyes faster to the left space than the right space after solving subtraction problems, while solving addition problems facilitates their eye movement to the right space. Moreover, it is found that gaze position shifts rightward during upward number counting, which confirms the hypothesis of a movement along the mental number line for addition (Hartmann et al., 2016).

If mental arithmetic operations are closely associated with space, the interaction between space and mental arithmetic could also occur in multiplication and division. Landy and Goldstone (2010) reported a spatial attention effect when solving symbolic arithmetic expressions involving addition and multiplication (e.g., “3 + 4 × 7”). They found that operands are more likely to be summed when they are widely spaced, and more likely to be multiplied when the operands are narrowly spaced (Landy and Goldstone, 2010). Consistently, Rivera and Garrigan (2016) replicated the study of Landy and Goldstone (2010) recently and confirmed that the physical spacing of formal equations has a large impact on successful evaluations of the expressions (Rivera and Garrigan, 2016). Katz and Knops (2014) were the first to explore the operational momentum effect in multiplication and division. They used a task in which a formula containing two operands and an operator were followed by five response choices. They found that in non-symbolic operations, adults preferred to select the option larger than the correct outcome for multiplication, and to select the option smaller than the correct outcome for division (Katz and Knops, 2014). However, these effects were not observed in symbolic multiplication and division, presumably because participants could calculate exactly and choose the correct values (Katz and Knops, 2014; Katz et al., 2017). Recently, contrasting results were reported by Shaki and Fischer (2017) who observed a reverse operational momentum effect with symbolic numbers. In this study, participants were asked to produce the line length matching the result of a symbolic arithmetic problem (multiplication or division). It was found that subjects produced larger outcomes than a baseline value in division, and no difference between outcomes and the baseline in multiplication. They proposed that the reverse operational momentum effect in division reflects strong anchoring on the large first operands in division compared with multiplication (Shaki and Fischer, 2017).

**FIGURE 1:**
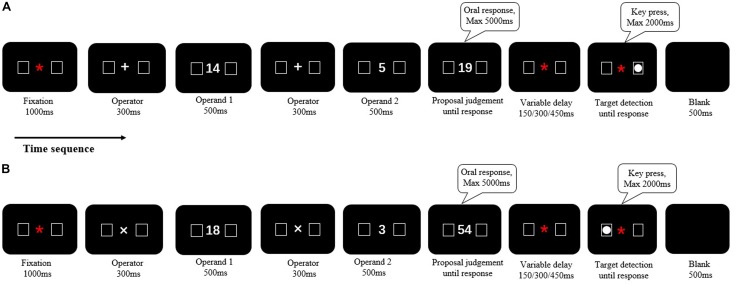
The timing and task sequence of an example trial. The fixation, operator, first operand, operator, second operand, and the proposal answer were presented successively at the center of the screen. The proposal would not disappear until a verbal response with a maximum duration of 5000 ms. The target (a solid circle, white) was randomly presented either in left- or in right-side box for 80% of the trials. It would disappear as soon as participant response with a maximum duration of 2000 ms. There was a variable delay (150, 300, or 450 ms) between the proposal and target. **(A)** It was for addition and subtraction (Experiment 1), and **(B)** it was for multiplication and division (Experiment 2). The signs of “^∗^, +, ×” mean fixation, addition and multiplication, respectively.

Although these studies have provided some evidence for a spatial-arithmetic association in multiplication or division, such work remains scarce, and the conclusions from earlier studies are controversial. In order to make a complete case for the interaction between space and mental arithmetic, it is worth considering whether multiplication or division operation can induce shifts of spatial attention similar to those found in addition and subtraction operations (Masson and Pesenti, 2014; Mathieu et al., 2016; Liu et al., 2017a,b). In the present study, we used symbolic magnitudes (Arabic numerals) to investigate shifts of spatial attention not only in addition and subtraction, but also in multiplication and division with a within-subject design. We adopted the target detection paradigm of Liu et al. (2017a), in which participants should perform two tasks consecutively: (1) the mental arithmetic task, in which they must solve an arithmetic problem and provide an oral judgment on whether a proposed result was correct or incorrect; (2) the target detection task, in which they are required to decide whether the target stimulus (a solid white circle) was presented or not (see **Figure 1**). It has been demonstrated that shifts of spatial attention caused by simple number processing (e.g., Fischer et al., 2003), as well as by mental arithmetic (e.g., Mathieu et al., 2016; Liu et al., 2017a), are strongly time-dependent. Thus, between the mental arithmetic task and target detection, three variable delays (150, 300, and 450 ms) were introduced to investigate the time course of any potential shifts of attention induced by arithmetic operations.

Current theories of arithmetic processing make different predictions for the interaction between space and mental arithmetic. According to the neural computational model of Chen and Verguts (2012), addition and subtraction operations recycle the radial basis function network for spatial transformations by a compressed mapping between numbers and space. The basis function network, corresponding to multimodal parietal areas such as LIP (lateral intraparietal area) and VIP (ventral intraparietal area), is used for saccadic and attentional control (Knops et al., 2009a), and plays a key role for numerical arithmetic (Chen and Verguts, 2012). Thus, the neural computational model provides a potential explanation for the shifts of spatial attention in addition and subtraction operations. However, the classical computational model (Verguts and Fias, 2005) suggests that multiplication problems are solved primarily by memory retrieval instead of a spatial transformation for addition and subtraction operations. Thus, based on these computational models, we did not predict that the spatial-arithmetic association would be observed in multiplication and division operations. In contrast, an alternative theory proposes that the space-arithmetic interaction might instead be due to semantic associations, such as “left-small,” “right-large,” “left-minus,” or “right-plus” (Gevers et al., 2010; Masson and Pesenti, 2014; Hartmann et al., 2015; Winter et al., 2015; Mathieu et al., 2016). Based on this account, one can predict that rightward shifts of spatial attention might be observed both in addition and multiplication, and a leftward shift of spatial attention in subtraction and division, because the former two operations make outcomes larger and the latter two operations make outcomes smaller for positive integers.

## Materials and Methods

### Experiment 1

We first examined the interaction between space and arithmetic in addition and subtraction operations, i.e., whether solving addition problems would accelerate the detection of right-side targets, and solving subtraction problems would facilitate the detection of left-side targets.

#### Participants

Based on our previous study (Liu et al., 2017a), the required sample size should be 27 subjects to achieve a power of 95%. In the present study, 27 students (12 males, all right-handed) were recruited with an age range from 18 to 24. All participants had normal or corrected-to-normal vision and were naïve with respect to the objective of this study.

The present study was approved by the Human Research Ethics Committee for Non-Clinical Faculties, School of Psychology, South China Normal University. The procedures and other relevant details of the experiments were in accordance with the approved guidelines as well as the ethical guidelines. We obtained informed consent from all subjects before the experiments.

#### Materials and Design

The arithmetic problems used in Experiment 1 were identical to those in Liu et al. (2017a), Experiment 1. There were 360 trials in Experiment 1. These trials were divided into 4 blocks; each block had 90 trials. All stimuli can be seen in Supplementary Table S1.

#### Task and Procedure

Stimuli were displayed on a Lenovo PC equipped with a 23-inch screen. Stimulus presentation and data collection were programmed using E-prime 2.0 software.

The sequence of an example trial was as follows (see **Figure 1A**). At first, a red fixation “^∗^” (Calibri 20 pt, 4 mm, 0.4°) was presented in the center of the screen with two lateral boxes (each with 4.5° eccentricity, 1 cm × 1 cm, 1° × 1°) for 1000 ms. One was at the left of the fixation and the other was at the right. As soon as the fixation disappeared, an operator (“+” or “-”) (Calibri 40 pt, 8 mm, 0.8°) appeared in the center of the screen for 300 ms, indicating the following arithmetic operation. Then, the first operand (500 ms), operator (300 ms), and second operand (500 ms) appeared in the center of the screen successively. Each digit was in Calibri 36 pt, 1.1 cm × 0.6 cm, 1° × 0.6°. After this, a proposal was presented in the center of the screen, and participants were asked to give an oral judgment [“Dui (Yes)” for correct results and “Cuo (No)” for incorrect results] as quickly and accurately as possible. The proposal remained on the screen until a response was made or for a maximal duration of 5000 ms. After the proposal disappeared, a variable delay (150, 300, or 450 ms) was introduced and followed by a target (a solid white circle, 0.7° diameter) presented either in the right or in the left box. Participants were instructed to press the space bar with their right hand as soon as they detected the target within a duration of 2000 ms. In 80% of the trials, the targets appeared randomly in the left or right box. The other 20% trials were catch trials in which no target appeared. These trials were introduced to prevent anticipatory responses.

Before testing, participants were informed that the mental arithmetic task had no relation with the target detection task, and that they should keep their eyes fixated on the center of the screen during the whole experiment. There were 16 practice trials before the experiment for each participant.

#### Data Analysis and Results

Trials with errors in either mental arithmetic task or target detection task were excluded from further analysis (7.35%). Moreover, the following trials were also discarded: (1) in the arithmetic task, trials in which the microphone failed to trigger or the reaction time of the oral response was more than 5000 ms (5.47%); (2) in the target detection task, trials in which the reaction time for detecting the target was an outlier (more than three standard deviations away from the mean; 1.88%). In both experiments, we only report the results of the target detection task. The full list of mean RTs (and SD) as a function of operation, target side and delay in two experiments are presented in **Table 1**.

**Table 1:** Mean RT (and SD) of the target detection task as a function of Arithmetic, Target side, and Delay (in ms) in two experiments.

Experiment 1	Addition			Subtraction		
	150	300	450	150	300	450
left	420	384	385	413	377	382
	(88)	(96)	(82)	(76)	(77)	(76)
right	412	370	373	422	391	384
	(90)	(88)	(84)	(91)	(83)	(82)

**Experiment 2**	**Multiplication**			**Division**		
	**150**	**300**	**450**	**150**	**300**	**450**

left	416	371	376	425	381	383
	(80)	(73)	(63)	(65)	(71)	(68)
right	406	366	371	419	385	374
	(71)	(69)	(59)	(74)	(83)	(64)

**FIGURE 2:**
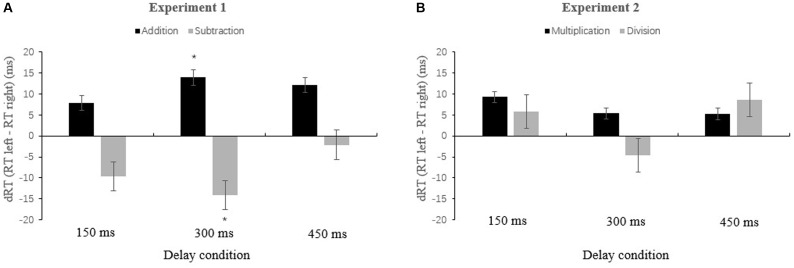
The difference in dRT as a function of arithmetic (addition, subtraction, multiplication, and division) with 150, 300, and 450 ms delay in two experiments. **(A)** It was for addition and subtraction (Experiment 1), and **(B)** it was for multiplication and division (Experiment 2). dRT = RT (left) - RT (right). “Up” bars mean that the targets presented in the right-side box are detected faster than those in the left-side box, while “down” bars mean that left-side targets are detected faster than right-side targets. Error bars represent SEM. ^∗^*p* < 0.05; ^∗∗^*p* < 0.001. Here and elsewhere, we displayed only the target detection data (see main text).

A 2 × 2 × 3 repeated-measures analysis of variance (ANOVA) was first carried out with arithmetic operation (addition or subtraction), target side (left or right), and delay (150, 300, or 450 ms) as within-subject factors (see **Figure 2A**). We found a main effect of delay, *F*(2,52) = 51.62, *p* = 0.001, *η*^2^ = 0.665. Mean RTs were the fastest in the 300 ms delay condition, and were significantly faster than the 150 ms delay condition, *F*(1,26) = 49.711, *p* = 0.001, *η*^2^ = 0.657. There was no significant difference between the 300 ms and 450 ms delay conditions, *F*(1,26) = 0.012, *p* = 0.65, *η*^2^ = 0.001. Most relevant for our study, there was a significant interaction between arithmetic operation and target side, *F*(1,26) = 16.843, *p* = 0.001, *η*^2^ = 0.393. An examination of the simple effect of side after each type of problem showed that participants detected right-side targets faster than left-side after addition problems, *F*(1,26) = 10.317, *p* = 0.003, *η*^2^ = 0.284. In contrast, after subtraction problems, they detected left-side targets faster, *F*(1,26) = 12.297, *p* = 0.03, *η*^2^ = 0.321. The interaction of arithmetic operation × target side × delay was not significant, *F*(1,26) = 1.214, *p* = 0.305, *η*^2^ = 0.045. There were no other main or interaction effects.

Second, in order to reveal the time course of spatial-arithmetic association, we conducted a series of pairwise *t*-tests for the different delay conditions. The results showed that, after solving addition problems, participants detected right-side targets faster than left-side targets only in the 300 ms delay condition, *t*_26_ = 2.286, *p* = 0.031. In the 450 ms delay condition, there was a marginally significant effect, *t*_26_ = 1.992, *p* = 0.057. For subtraction problems, left-side targets were detected significantly faster than right only in 300 ms delay condition, *t*_26_ = 2.262, *p* = 0.032. A *post hoc* test for multiple comparisons further indicated that at 150 ms delay condition, there was no significant difference between right-side and left-side targets for both addition and subtraction operations; at 300 ms delay condition, the spatial-arithmetic association effect was robust (significant, *p* = 0.031 for addition and *p* = 0.032 for subtraction), but growing weaker at 450 ms (marginally significant for addition, *p* = 0.057; but non-significant for subtraction, *p* = 0.723).

Given that in the target detection task stimuli were triggered by oral responses from the mental arithmetic task, it is possible that there were trade-offs between the proposal judgment RTs and target detection RTs. We therefore checked the correlation between individual-trial proposal judgment RTs and target detection RTs (see Supplementary Figure S1). Statistical analysis (with subject as a random effect) revealed a small and non-significant correlation between the two RTs (*r* = 0.144; *p* = 0.638).

### Experiment 2

In this experiment, we investigated whether multiplication and/or division can induce covert spatial attention shifts similar to those found following addition and subtraction operations.

#### Participants

Participants in Experiment 2 were the same as in Experiment 1. The order of the two experiments was counterbalanced: half of the subjects took part in Experiment 1 first and then Experiment 2 about 2 days later; the other half of the subjects took part in Experiment 2 first and then Experiment 1 after a similar time interval.

#### Stimuli and Design

The arithmetic problems for the multiplication and division operations were created according to the criteria of Katz and Knops (2014) (see Supplementary Table S2). The magnitudes of the proposal arithmetic results were matched for multiplication and division. For each correct result (*c*), four possible incorrect results were generated using a geometric series: *c* × 1.5*^i^*^/3^ (*i* from -3 to 3). To control the parity, the deviant results were rounded to the closest value with the same parity as the correct results. We also discarded the two most extreme values because they were too far away from the correct results to be plausible (Katz and Knops, 2014).

#### Task and Procedure

The task and procedure were same as that in Experiment 1, except that the operator signs (“ × ” or “÷”), operands and proposed results values were changed.

#### Data Analysis and Results

We used the same exclusion criteria for data as in Experiment 1. Here, 7.73% error and extreme trials were excluded from the analysis. A 2 × 2 × 3 repeated-measures ANOVA was conducted with arithmetic operation (multiplication or division), target side (left or right), and delay (150, 300, or 450 ms) as within-subject factors. The results showed that there was a main effect of arithmetic operand, *F*(1,26) = 25.758, *p* = 0.001, *η*^2^ = 0.479. The mean RTs for target detection following multiplication were significantly faster than following division. The main effect of delay was also significant, *F*(1,26) = 70.169, *p* = 0.001, *η*^2^ = 0.715. Mean RTs were fastest in the 300 ms delay condition, which was significantly faster than in the 150 ms delay condition, *F*(1,26) = 74.911, *p* = 0.001, *η*^2^ = 0.728, but there was no difference from the 450 ms delay condition, *F*(1,26) = 0.001, *p* = 0.921, *η*^2^ = 0.001. Most important for our results, we did not find the hypothesized interaction between arithmetic operation and target side, *F*(1,26) = 0.771, *p* = 0.388, *η*^2^ = 0.027; and the three-way interaction of arithmetic operation, target side and delay, *F*(1,26) = 1.248, *p* = 0.295, *η*^2^ = 0.043. Further pairwise *t*-tests indicated no difference between the mean RTs for detection following multiplication and division operations in any delay condition (see **Figure 2B**). The *post hoc* test showed that the detection time for right-side target were not significantly different from those for left-side targets in all three delay conditions (150 ms: *p* = 0.093 for multiplication and *p* = 0.154 for division; 300 ms: *p* = 0.259 for multiplication and *p* = 0.378 for division; 450 ms: *p* = 0.218 for multiplication and *p* = 0.081 for division).

The correlation between individual-trial proposal judgment RTs and target detection RTs were also examined for Experiment 2 (see Supplementary Figure S1). Statistical analysis (with subject as a random effect) revealed a small and non-significant correlation between the two RTs (*r* = 0.134; *p* = 0.615).

### Comparison the Spatial-Arithmetic Association Effects Between Experiment 1 and Experiment 2

Finally, in order to quantify the strength of the interaction between arithmetic operations and target side, Bayesian statistics for null-hypothesis significant testing (NHST) were conducted for both Experiments (Raftery, 1995; Masson, 2011). The results showed that the posterior probability value of the alternative hypothesis for Experiment 1 was 0.984 and the Bayes factor was 61.941, which indicated that there was strong evidence for an interaction between operation (addition, subtraction) and target side (right, left) (Raftery, 1995; Masson, 2011). In experiment 2, however, the posterior probability value of the null hypothesis was 0.782 and its Bayes factor was 3.591 (Raftery, 1995; Masson, 2011), suggesting that there was no clear evidence for an interaction between arithmetic operation (multiplication, division) and target side (right, left).

## Discussion

In the current study, we investigated the interaction between space and arithmetic for addition and subtraction operations in Experiment 1, and for multiplication and division operations in Experiment 2. The results indicated that after solving addition problems targets on the right side were detected faster than those on the left side, while left-side targets were detected faster than those on the right side after solving subtraction problems. However, we did not find enough evidence for this space-arithmetic interaction effect in multiplication or division.

Our findings in addition and subtraction operations confirmed the hypothesis that addition and subtraction operations could induce horizontal shifts of spatial attention (e.g., Masson and Pesenti, 2014; Mathieu et al., 2016; Masson et al., 2017; Liu et al., 2017a). In line with the prediction based on the neuro-computational model of Chen and Verguts (2012), the interaction between space and arithmetic operation of Experiment 1suggested that a neural network for spatial transformations was recycled during mental addition and subtraction. It was also consistent with the findings from brain-imaging studies, which have indicated that addition (and subtraction) operations recruit neural structures that support the orienting of spatial attention (Dehaene et al., 2003; Knops et al., 2009a; Mathieu et al., 2017).

Based on the “semantic association theory,” the shifts of spatial attention would be present in addition and subtraction operations, as well as in multiplication and division. However, we found no clear evidence for a spatial-arithmetic association in multiplication or division. At the very least, the results in the present study showed that the interaction between space and multiplication (or division) operation was – if it exists at all – weaker than those for addition and subtraction operations. These results also support the findings of Katz et al. (2017) who found that the cognitive processes underlying multiplication and division are less prone to spatial biases compared to addition and subtraction. Therefore, the dissociation in our current study suggested that the cognitive mechanisms underlying multiplication and division operations might be different from those for addition and subtraction (Katz et al., 2017). However, the Bayes factor analysis indicated that more data are needed to make this conclusive in future work.

It has been reported that the neural activity during multiplication is consistent with performance based on verbal processing, and qualitatively different from addition which would rely more strongly on visuospatial processing (Zhou et al., 2007). In trained multiplication (relative to untrained multiplication), a significant focus of activation appeared in the left angular gyrus, which has been observed in other studies assessing arithmetic fact retrieval (Gruber, 2001; Ischebeck et al., 2006). Recently, a functional MRI study found during trials containing only an addition sign, a significant correlation between the frontal eye field (FEF) and the posterior superior parietal lobule (PSPL). In contrast, for trials containing only a multiplication sign, no such correlation was found (Mathieu et al., 2017). Together, these findings demonstrate that multiplication and division recruit different neural networks from addition and subtraction (Mathieu et al., 2017). Moreover, it has been suggested that single-digit multiplication performance is dominated by retrieval of multiplication tables from memory (Verguts and Fias, 2005). It was plausible that the null effect of spatial-arithmetic association in multiplication or division was caused by its exact answer instead of approximate arithmetic which has a functional association with spatial attention (Katz et al., 2017).

## Conclusion

We investigated for the first time the relation between all four arithmetic operations (addition, subtraction, multiplication, and division) and space. Our results confirmed the presence of space-arithmetic interaction for addition and subtraction operations, but there was no clear evidence for a similar interaction effect in multiplication or division. These differences might be due to fundamental differences of addition and subtraction from multiplication and division: for addition and subtraction, the spatial-arithmetic association is attributed to the mapping between the numerical representation of operands and space, and based on a basis function network for spatial transformation (Chen and Verguts, 2012), whereas multiplication and division may consist of the retrieval of a multiplication table from memory (Mathieu et al., 2017).

## Author Contributions

QC conceived and supervised the experiments. QC, MeL and DL designed the experiments. MeL, DL, MiL, WD and YH implemented the experiments and collected data. MeL and DL analyzed the results. MeL, DL, MiL, WD, YH and QC wrote and revised the paper.

## Conflict of Interest Statement

The authors declare that the research was conducted in the absence of any commercial or financial relationships that could be construed as a potential conflict of interest.
