# Supplementary material for: Addition and Subtraction but Not Multiplication and Division Cause Shifts of Spatial Attention
Source: Front Hum Neurosci. 2018 May 3;12:183. doi: 10.3389/fnhum.2018.00183 (PMC5943508; doi:10.3389/fnhum.2018.00183)

**Supplementary Materials**

**Addition and Subtraction but not Multiplication and Division Cause Shifts of Spatial Attention**

Mengjin Li^1, 2, 3+^, Dixiu Liu^1, 2, 3, 4+^, Min Li^1, 2, 3^, Wenshan Dong^1, 2, 3^, Yalun Huang^1, 2, 3^, Qi Chen^1, 2, 3*^

^1^School of Psychology, South China Normal University, 510631 Guangzhou, China

^2^Center for Studies of Psychological Application, South China Normal University,

510631 Guangzhou, China

^3^Guangdong Key Laboratory of Mental Health and Cognitive Science, South China

Normal University, 510631 Guangzhou, China

^4^Department of Psychology, Gannan Medical University, 341000 Ganzhou, China

^+^Mengjin Li and Dixiu Liu have contributed equally to this work.

Running head: Arithmetic induces spatial attention shifts

Correspondence: Qi Chen

School of Psychology

South China Normal University

510631 Guangzhou

P.R. China

Email: [chen.qi@m.scnu.edu.cn](mailto:chen.qi@m.scnu.edu.cn)

**Table S1**

All Arithmetic Problems Presented in Experiment 1 and Their Correct and Deviant Results.

| Operands | |  | Proposals (Correct×2^i/4^） | | | | |
| --- | --- | --- | --- | --- | --- | --- | --- |
| O1 | O2 |  | -2 | -1 | 0 | 1 | 2 |
| Addition | | | | | | | |
| 14 | 5 |  | 13 | 16 | 19 | 23 | 27 |
| 14 | 7 |  | 15 | 18 | 21 | 25 | 30 |
| 14 | 11 |  | 18 | 21 | 25 | 30 | 35 |
| 28 | 7 |  | 25 | 29 | 35 | 42 | 49 |
| 28 | 13 |  | 29 | 34 | 41 | 49 | 58 |
| 28 | 21 |  | 35 | 41 | 49 | 58 | 69 |
| 56 | 13 |  | 49 | 58 | 69 | 82 | 98 |
| 56 | 28 |  | 59 | 71 | 84 | 100 | 119 |
| 56 | 42 |  | 69 | 82 | 98 | 117 | 139 |
| Subtraction | | | | | | | |
| 32 | 13 |  | 13 | 16 | 19 | 23 | 27 |
| 32 | 11 |  | 15 | 18 | 21 | 25 | 30 |
| 32 | 7 |  | 18 | 21 | 25 | 30 | 35 |
| 64 | 29 |  | 25 | 29 | 35 | 42 | 49 |
| 64 | 23 |  | 29 | 34 | 41 | 49 | 58 |
| 64 | 15 |  | 35 | 41 | 49 | 58 | 69 |
| 128 | 59 |  | 49 | 58 | 69 | 82 | 98 |
| 128 | 44 |  | 59 | 71 | 84 | 100 | 119 |
| 128 | 30 |  | 69 | 82 | 98 | 117 | 139 |

**Table S2**

All Arithmetic Problems Presented in Experiment 2 and Their Correct and Deviant Results.

| Operands | |  | Proposals (Correct×1.5^i/3^） | | | | |
| --- | --- | --- | --- | --- | --- | --- | --- |
| O1 | O2 |  | -2 | -1 | 0 | 1 | 2 |
| Multiplication | | | | | | | |
| 12 | 2 |  | 18 | 22 | 24 | 28 | 32 |
| 14 | 2 |  | 22 | 24 | 28 | 32 | 36 |
| 16 | 2 |  | 24 | 28 | 32 | 36 | 42 |
| 12 | 3 |  | 28 | 32 | 36 | 42 | 48 |
| 14 | 3 |  | 32 | 36 | 42 | 48 | 56 |
| 12 | 4 |  | 36 | 42 | 48 | 54 | 62 |
| 17 | 3 |  | 39 | 45 | 51 | 57 | 67 |
| 13 | 4 |  | 38 | 46 | 52 | 58 | 68 |
| 19 | 3 |  | 43 | 49 | 57 | 63 | 73 |
| Division | | | | | | | |
| 72 | 3 |  | 18 | 22 | 24 | 28 | 32 |
| 84 | 3 |  | 22 | 24 | 28 | 32 | 36 |
| 96 | 3 |  | 24 | 28 | 32 | 36 | 42 |
| 108 | 3 |  | 28 | 32 | 36 | 42 | 48 |
| 84 | 2 |  | 32 | 36 | 42 | 48 | 56 |
| 96 | 2 |  | 36 | 42 | 48 | 54 | 62 |
| 102 | 2 |  | 39 | 45 | 51 | 57 | 67 |
| 104 | 2 |  | 38 | 46 | 52 | 58 | 68 |
| 114 | 2 |  | 43 | 49 | 57 | 63 | 73 |

**Figure S1**

All individual oral latencies (Judgment RT) against target detection times

(Detection RT) in different SOA (150 ms, 300 ms and 500 ms) for Experiment 1 and Experiment 2.


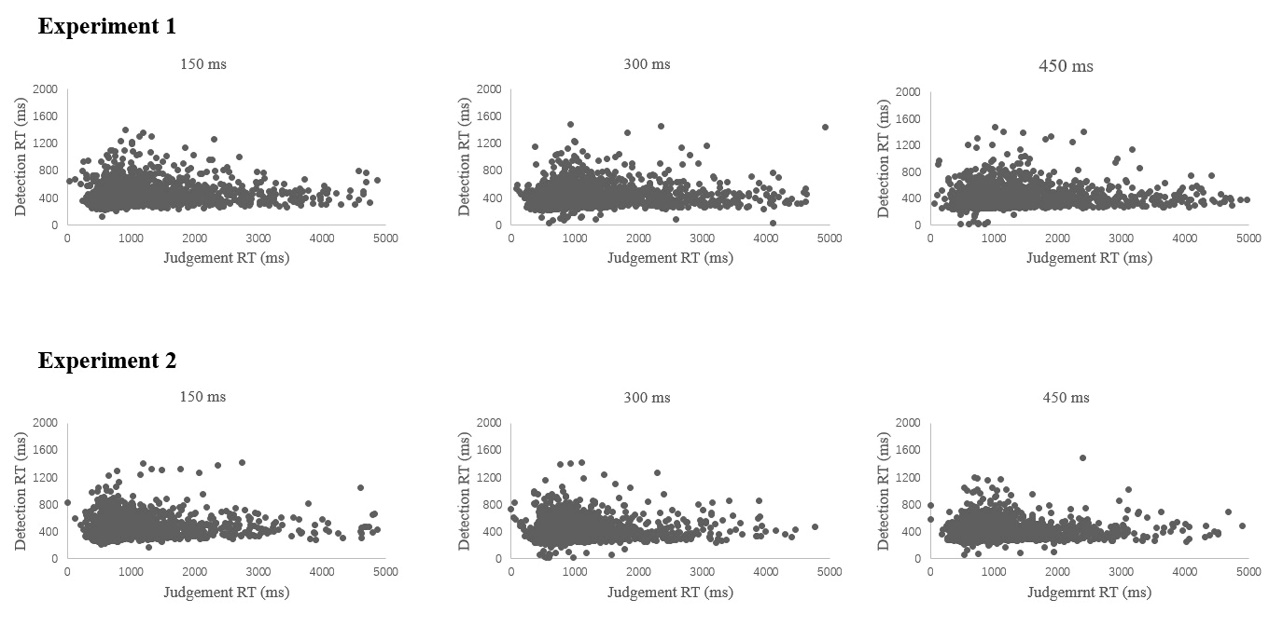

Supplement: Supplementary file 1 [file Data_Sheet_1.doc]
